# Prognostic value of venous blood analysis at the start of CPR in non-traumatic out-of-hospital cardiac arrest: association with ROSC and the neurological outcome: do not forget the no-flow influence!

**DOI:** 10.1186/s13054-020-02898-3

**Published:** 2020-05-18

**Authors:** Romain Jouffroy, Benoît Vivien

**Affiliations:** SAMU de Paris, Service d’Anesthésie Réanimation, Hôpital Universitaire Necker - Enfants Malades, AP-HP. Centre, and Université de Paris, Paris, France

To the Editor:

Corral Torres et al. [1] observed a significant relationship between severe alterations of venous blood gas variables and potassium at the start of cardiopulmonary resuscitation (CPR) of non-traumatic out-of-hospital cardiac arrest (OHCA) and low return of spontaneous circulation (ROSC) rate and neurological prognosis. The authors should be congratulated for their noteworthy study on this relevant question; nevertheless, we believe that the results interpretation requires words of caution because of some issues.

Firstly, the authors do not report major confounders of venous blood analysis, especially pre-analytical conditions. For example, during CPR, it is often common to encounter intravenous line insertion difficulties [2], involving a longer low-flow period justifying intraosseous access [3]. Consequently, by itself, it may induce acidaemia and hyperkalaemia.

Secondly, it is not reported if the result of blood gas venous analysis was taken into account in the care-delivered decision-making. For example, it is not reported how hyperkalaemia treatment was considered and/or treated and if the SAMUR (SAMUR: *Servicio de Asistencia Municipal de Urgencia y Rescate*, i.e. the specialized emergency system of Madrid, Spain) team physician decision was not influenced by the results’ analysis (e.g. care discontinuation because major blood gas analysis abnormalities).

Thirdly, from a methodological point of view, the variables included in the multivariate analysis (age, sex, first monitored rhythm, witnessed cardiac arrest, previous manoeuvres) do not consider the in-hospital phase confounders for neurological outcome [4] and the no-flow duration [5].

Therefore, we believe that the egg and the chicken should not be confused: the longer no-flow duration, the greater metabolic consequences will be marked. Beyond this, the authors’ work is very interesting, but more information and conclusions could be drawn by, first, integrating the no-flow duration in the analysis and, second, using the metabolic trend changes occurring during CPR.

## Authors’ response

Ervigio Corral Torres, Alberto Hernández-Tejedor

We thank Drs. Jouffroy and Vivien from SAMU, with whom we shared so much, for their letter to our recent article regarding the prognostic value of blood analysis at the start of CPR in non-traumatic out-of-hospital cardiac arrest [1]. Firstly, whatever the difficulties in obtaining the first venous line, the truth is that it is the first sample available in every real situation. Internal data have shown that the mean and average times do not exceed 70 s, which are in line with international recommendations [6]. Anyway, it cannot be completely ruled out in individual cases.

Secondly, since it was a pragmatic study, the physician decisions were based on our service’s procedures, which are public [7]. As far as we know, there were no previous studies about the prognostic value of blood gas analysis at the beginning of advanced out-of-hospital CPR, so it is unlikely that decisions could have been influenced by these results. In fact, trying to obtain cut-off points with a predictive value for decision-making, if it can be achieved, it would be a later study.

Thirdly, the main reason for not considering the no-flow time is because we never found time data from phone calls to be reliable enough. In fact, we really intended to find a different decision-making tool that helps the physician on scene, some kind of metabolic watch.

We appreciate the authors’ kind opinion on of our study. We agree on the interest that the metabolic trend changes will have. In fact, we have been working on it and hope to publish the results soon.

## Data Availability

Not applicable
